# Improving energy performance of buildings: Dataset of implemented energy efficiency renovation projects in Latvia

**DOI:** 10.1016/j.dib.2023.109225

**Published:** 2023-05-09

**Authors:** Elissaios Sarmas, Maria Kleideri, Aija Zučika, Vangelis Marinakis, Haris Doukas

**Affiliations:** a Decision Support Systems Laboratory, School of Electrical & Computer Engineering, National Technical University of Athens, Greece; b The Latvian Environmental Investment Fund (LEIF), Riga, Latvia

**Keywords:** Energy efficiency, Renovation measures, Investment financing, Gas emission reduction, Renewable energy sources

## Abstract

This article includes data collected from public and private buildings in Latvia as part of several projects/tenders funded from the governmental Climate Change Financial Instrument (KPFI^1^) of Republic of Latvia. The data provided consists of information on 445 projects, the activities performed therein, as well as numerical data on CO_2_ emission and energy consumption before and after the projects’ implementation. Data cover a period from 2011 to 2020 for various types of buildings. Given the amount, the completeness and the accuracy of data accompanied by qualitative and quantitative information on the funded projects, the datasets could be relevant for evaluating the energy efficiency of the implemented activities and the levels of CO_2_ and energy reduction. The reported figures could be used for further research on the field of energy performance of buildings and buildings’ refurbishments. They could be also taken as case studies by other buildings that plan to implement similar actions.

**Specifications Table**

**Table:** 

Subject	Energy

Specific subject area	Energy consumption and CO_2_ emission of buildings, energy efficiency activities, building data, project costs and grants data
Type of data	Data in Excel sheets (.xlsx). Tables and Figures (.jpeg)
How the data were acquired	All objects of the dataset received co-financing for renovation. Energy consumption before renovation, building information and investment amount is received from project applicants. Data after renovation is collected directly from buildings stakeholders. Data is collected in five years monitoring period after project implementation, in accordance to the agreement that the financial recipient had after the project monitoring period. Planned project results must be achieved during that monitoring period.
Data format	Raw, pre-processed and partly analysed data in pivot tables.
Description of data collection	All data has been provided by stakeholders at an annual basis during the monitoring period. Stakeholders collected measured energy consumption. Energy consumption was adjusted due to climatic conditions to compare it with planned results.
Data source location	• Institution: The Latvian Environmental Investment Fund • City/Town/Region: Riga • Country: Latvia • Latitude and longitude (and GPS coordinates, if possible) for collected samples/data: 56.961458091377395, 24.09865005412116 • All data collected by stakeholders were sent to The Latvian Environmental Investment Fund.
Data accessibility	Repository name: Mendeley Data Data identification number: 10.17632/x6wyhmpj2v.2 Direct link to data: *https://data.mendeley.com/datasets/x6wyhmpj2v/2*
Related research article	*Sarmas, E., Spiliotis, E., Marinakis, V., Koutselis, T., & Doukas, H. (2022). A meta-learning classification model for supporting decisions on energy efficiency investments. Energy and Buildings, 258, 111836*. *https://doi.org/10.1016/j.enbuild.2022.111836*

## Value of the Data

• Data provide valuable information on the implementation of funded projects for the improvement of buildings’ energy efficiency [2]. They can be relevant for the evaluation of a variety of activities taken place in different types of buildings, aiming at the reduction of the buildings’ CO_2_ emission and energy consumption [3].
• Researchers, policy makers, as well as professionals working for the improvement of energy performance in buildings [4,5] may benefit from the data collected under the framework of the KPFI government programme.
• Data can be used / reused for performing further analysis of the given variables, assessing the efficiency of the implemented renovation actions, comparing energy indicators before and after implementation and exploiting the projects results in order to initiate similar projects/activities in the future [6].

## 1 Objective

Data presented in this article are collected from a list of projects implemented in Latvia and funded by the government Climate Change Financial Instrument (KPFI) of Republic of Latvia. The general scope of this funded programme was to provide the motivation and the funds to stakeholders in order to implement renovation activities on buildings that would result in the improvement of the buildings’ energy performance, the development and implementation of technologies that would use renewable energy resources, as well as the implementation of integrated solutions to reduce greenhouse gas emissions. To this respect, the data collected through the monitoring process of these projects are provided in this article with the aim of sharing the projects’ results for further research and the assessment of the actual implementation of such renovation activities in buildings.

## 2 Data Description

The present article refers to a set of datasets collected from the aforementioned projects in various buildings in Latvia. The main variables included in the datasets concern the buildings CO_2_ emissions (given in tons) and energy consumption (given in MWh) data along with relevant information on the particular projects and the buildings involved.

Data described in this article are available in Mendeley Data, DOI:10.17632/x6wyhmpj2v.2 [1] and are delivered in three datasets that are presented in detail below.

The first dataset ‘KPFI_Data on projects’ provides a list of the implemented projects with general information on their duration, costs and financing, the second dataset ‘KPFI_Data on activities’ includes the metadata for the buildings involved and the activities implemented during the projects while the third dataset ‘KPFI_Detailed implementation data’ concerns the detailed data collected through the projects’ implementation and monitoring. All three datasets include both the tender's and project's code number, variables that could be used as identification keys for the interconnection of the datasets and the combination of information and data in further research and analysis.

### 2.1 KPFI_Data on projects

this file includes general information on the implemented KPFI projects. It lists 445 projects that were implemented under 11 tenders of the funded KPFI programme of the Republic of Latvia.

The information provided in this first dataset includes the code number and name of tenders, the project code number, the CO_2_ reduction (in tons) as planned by each project, the start, end date and project duration (in months), the total costs and the grant financing of each project as well as the respective share of grant.

Coloured formatting is applied in the columns providing numeric information to facilitate the understanding and highlight the data ranges. In particular, coloured cells correspond to the maximum (marked blue) and minimum (marked yellow) value of each column. Additionally, a 2-colour scale is applied in all values of the share (%) of grant to highlight the distribution of shares, from lowest values (pale brown) to highest values (dark brown).

A summary of the total costs and grants received per KPFI project can be seen in the following table.

**Table 1:** Total costs and grants per KPFI tender.

Tender	Number of Projects	Total Project Costs (in millions Euro)	Total Grant Financing (in millions Euro)	% of Grant
KPFI-1_Increase of energy efficiency in municipal buildings (I round)	56	46.12€	31.69€	68.71%
KPFI-3_Increase of energy performance in higher education establishment buildings	13	11.46€	6.76€	58.99%
KPFI-5_Complex solutions for greenhouse gas emission reduction in state and municipal vocational education establishment buildings	23	16.69€	13.18€	79.01%
KPFI-6_Complex solutions for greenhouse gas emission reduction in manufacturing buildings	32	16.87€	6.72€	39.85%
KPFI-7_Complex solutions for greenhouse gas emission reduction in municipal buildings (II round)	38	32.29€	19.07€	59.05%
KPFI-10_Low energy consumption buildings	6	6.67€	3.36€	50.39%
KPFI-15_Complex solutions for greenhouse gas emission reduction (I round)	6	2.31€	0.71€	30.91%
KPFI-15.1_Complex solutions for greenhouse gas emission reduction (II round)	34	14.25€	6.62€	46.48%
KPFI-15.2_Complex solutions for greenhouse gas emission reduction (III round)	101	42.66€	18.18€	42.61%
KPFI-15.3_Complex solutions for greenhouse gas emission reduction (IV round)	95	37.97€	17.92€	47.18%
KPFI-15.4_Complex solutions for greenhouse gas emission reduction (V round)	41	14.88€	7.30€	49.06%

**Grand Total**	**445**	**242.17€**	**131.51€**	**54.31%**

### 2.2 KPFI_Data on activities

this file includes information on the renovation activities implemented in the KPFI projects.

The information provided thereon consists of the following variables: (a) The tenders’ code number, (b) the projects’ code number, (c) the building on which each project was implemented, (d) the detailed description of each implemented activity, (e) the CO_2_ reduction (in tons) per activity/building, (f) the energy reduction (in MWh) per activity/building and (g) the respective CO_2_ emission factor.

In addition, the dataset includes the type of building, marked from a list of 11 categories, i.e. college, higher education institution, cultural institution building, factory, hotel and other short-stay accommodation, medical institution, municipal administration building, office building, warehouse, etc. (companies), preschool educational institution, residential buildings (households), school, sports building and construction.

Finally, the activities implemented in the presented projects are grouped in 10 categories, provided as type of activity in the dataset. The activities listed are energy efficient lighting, energy sources with heat pumps, heat supply renovation, internal engineering networks, renovation of the building's enclosing structures, solar collector system, solar power plant, technological equipment, ventilation system renovation, wood chips, biomass pellets and straw boiler house.

**Fig. 1:**
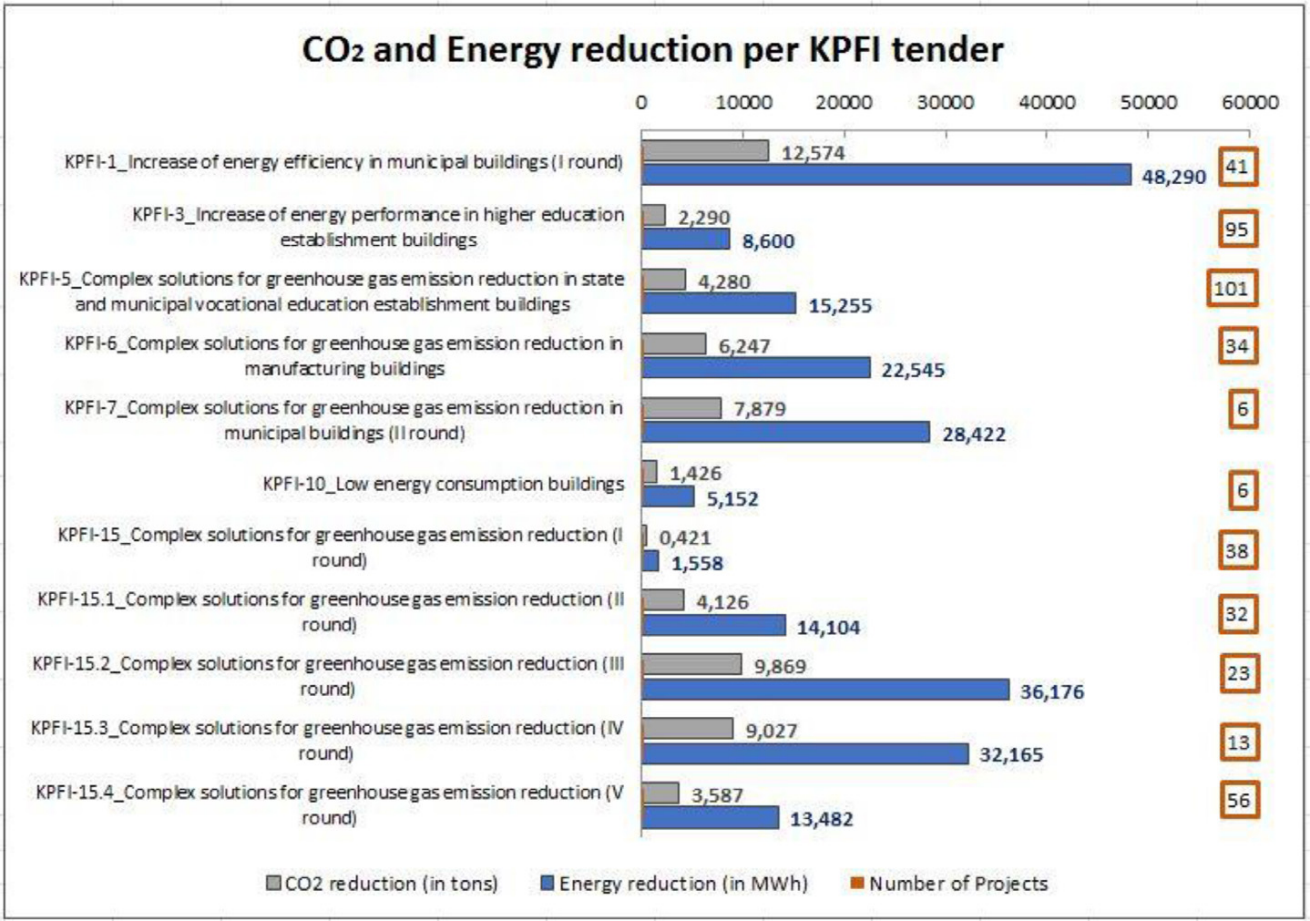
CO_2_ and energy reduction per KPFI tender.

**Fig. 2:**
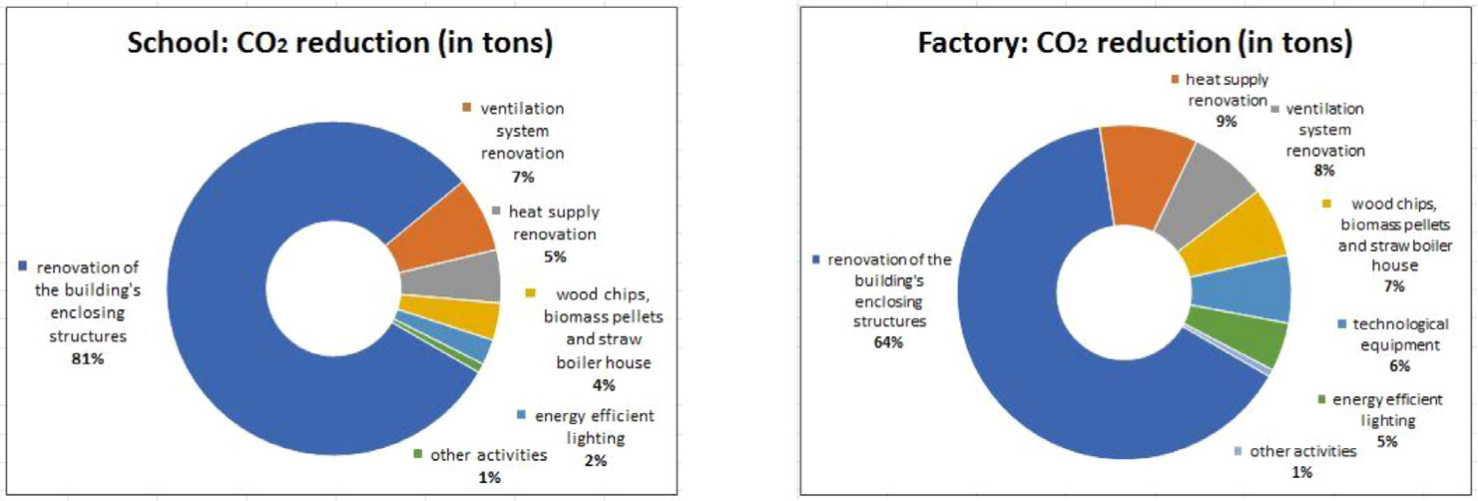
CO_2_ reduction per type of activity in schools and factories.

For a first understanding and presentation of the given data, the dataset is accompanied by a set of indicative pivot tables that provide a first insight on the data and how this could be combined and analysed. As part of this pre-analysis of the data, Table 2, Fig. 1, Fig. 2 are presented below.

**Table 2:** CO_2_ and energy reduction per type of activity implemented in KPFI tenders.

Type of activity	Number of activities in all buildings	CO_2_ reduction (in tons)	Energy reduction (in MWh)
Renovation of the building's enclosing structures	4442	46,149.989	171,405.774
Heat supply renovation	603	4316.539	16,100.424
Ventilation system renovation	413	4380.935	17,052.402
Energy efficient lighting	268	1869.098	5008.468
Wood chips, biomass pellets and straw boiler house	52	3607.650	11,613.983
Technological equipment	42	969.232	2671.415
Solar collector system	21	113.460	485.307
Energy sources with heat pumps	19	218.409	1150.010
Internal engineering networks	11	25.908	77.942
Solar power plant	1	72.794	183.360

**Grand Total**	**5872**	**61,724.014**	**225,749.085**

### 2.3 KPFI_Detailed implementation data

this file includes detailed information on all data collected through the monitoring process of the implemented projects. Data are provided for the five reporting years of each project, covering in total a period from 2011 to 2020.

The dataset consists of the following group of variables:

(a) General data information, including the reporting year, the tender's code number, the project's code number, the planned CO_2_ reduction per year, the number of houses in each project, the year of building, the number of floors, the total heating area (in m^2^), the house functions, the town and county of the buildings.
(b) Overall building data before project implementation, with reference to three sources of energy consumption, i.e., heating, hot water and electricity. The variables provided are: energy consumption before project (MWh), energy consumption before project (kWh/m^2^), CO_2_ emission before project (tons), meteorology station, source of heat (for heating), CO_2_ emission factor (tons CO_2_/MWh) (for heating), CO_2_ emission factor (audit) (tons CO_2_/MWh) (for heating), source of heat (for hot water), CO_2_ emission factor (tons CO_2_/MWh) (for hot water), CO_2_ emission factor (tons CO_2_/MWh) (for electricity).
(c) Detailed data on the project implementation, i.e., for each source of energy consumption (heating, hot water and electricity) the dataset provides information on the total energy consumption (in MWh) and the CO_2_ emission (in tons) before the project and at the reporting year of implementation. In each case, the reduction in energy consumption and in CO_2_ emission is also provided, as the difference between the values at the reporting year and before project implementation.
(d) The reported monthly data for the temperature, the heating days and the energy consumption (MWh) of each source.

**Fig. 3:**
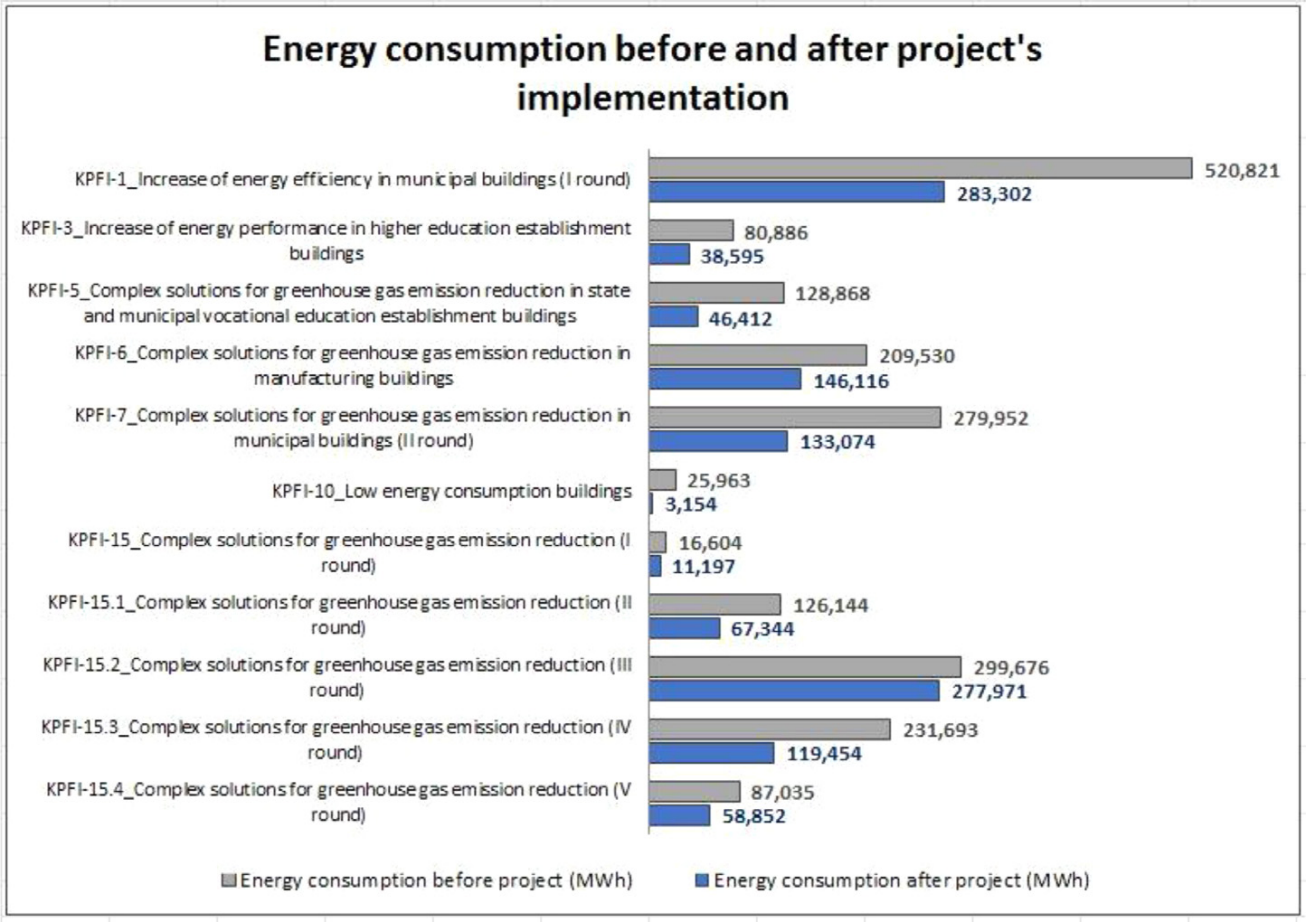
Total energy consumption before and after project's implementation.

Finally, the dataset is accompanied by an indicative pivot analysis for a first insight on the share of energy and CO_2_ reductions accomplished by each tender. The total energy consumption before and after implementation of the tenders can be also seen in Fig. 3 below.

## 3 Experimental Design, Materials and Methods

Data presented in this article are acquired from the Latvian Environmental Investment Fund in excel sheets, as provided to them by the stakeholders implementing the KPFI projects. Following the tenders’ financial agreement, the building stakeholders were required to report the measured energy data at an annual basis, covering a five-year monitoring period per project.

Data provided by stakeholders were thoroughly checked and validated where needed to ensure full data harmonisation. To this direction, given that data were reported from different stakeholders/buildings, a few edits and corrections were considered necessary to make sure that all numeric data are reported in a consistent way. Such edits mainly concerned the third dataset (KPFI_Detailed implementation data) and were applied following checks for (a) the reporting unit per variable (e.g. all figures of CO_2_ emissions given in tons), (b) the calculated differences (value at the reporting year minus value before implementation), (c) harmonisation of records/categories in the buildings’ information variables, (d) harmonised classification per type of activity and type of building. All performed checks/edits aimed at the release of a clean and harmonised set of datasets that could be fully exploited and re-used in future research and analysis. Data validation and processing/cleaning were completed without significant limitations and thus, the released datasets can be considered of high quality.

As regards the short analysis accompanied the three datasets, it was performed with the use of pivot tables and the addition of some ratios, calculated on the data pivot analysis. All processing and analysis of the data was made on Microsoft Office Excel, including the figures and tables presented above in the article Table 1.

## Ethics Statements

Data published within the present article, as well as the accompanied analysis, meet the Data in Brief ethical requirements for publication. It is clearly stated that the work carried out for the needs of the data collection and presentation does not involve human subjects, animal experiments, or any data collected from social media platforms. It should be further clarified that no personal data are included in the datasets concerning the participant buildings’ stakeholders and that the platform(s)’ data redistribution policies are fully complied with it.

## CRediT authorship contribution statement

**Elissaios Sarmas:** Conceptualization, Methodology, Writing – original draft. **Maria Kleideri:** Data curation, Visualization, Writing – original draft. **Aija Zučika:** Data curation, Investigation, Writing – original draft. **Vangelis Marinakis:** Methodology, Supervision, Writing – review & editing. **Haris Doukas:** Validation, Project administration, Writing – review & editing.

## Declaration of Competing Interest

The authors declare that they have no known competing financial interests or personal relationships that could have appeared to influence the work reported in this paper.

## Data Availability

Energy Efficiency Financing Dataset (Original data) (Mendeley Data).
